# Serum levels of soluble IL-2R, CD4 and CD8 in bronChial asthma

**DOI:** 10.1155/S0962935195000433

**Published:** 1995-07

**Authors:** G. Di lorenzo, P. Mansueto, M. Melluso, G. Morici, F. Norrito, D. Cigna, G. Candore, C. Caruso

**Affiliations:** 1 Istituto di Medicina Interna e Geriatria Università di Palermo Via del Vespro, 141 Palermo 90127 Italy; 2 Istituto di Patologia Generale Università di Palermo Corse Tukory 21 1 Palermo 90134 Italy

## Abstract

The aim of the present study was to compare serum levels of soluble forms of interleukin-2 receptor, CD4 and CD8, released by lymphocytes during activation of the immune system, in patients with allergic bronchial asthma, with those in healthy subjects. Significantly higher levels of soluble IL-2R and soluble CD4 were found in patients with asthma compared with the control group. In contrast, lower levels of soluble CD8 values were found in patients with asthma compared to the control group. Significant correlations were found for both sIL-2R and sCD4 and these two molecules, with lung function measured as bronchial responsiveness to inhaled methacholine. These results strengthen previous suggestions that in allergic bronchial asthma, activation of T cells plays a significant role in the disease pathogenesis.


Mediators of Inflammation 4, 270–272 (1995)

### Serum levels of soluble IL-2R, CD4 and CD8 in bronchial asthma

G. Di Lorenzo,[1] P. Mansueto,[1] M. Melluso,[1] G. Morici,[1] F. Norrito,[1] D. Cigna,[2] G. Candore[2] and C. Caruso[2,CA]

[1]Istituto di Medicina Interna e Geriatria, Università di Palermo, Via del Vespro, 141, 90127 Palermo, Italy; [2]Istituto di Patologia Generale, Università di Palermo, Corso Tukory 211, 90134 Palermo, Italy.

[CA]Corresponding Author

**The aim of the present study was to compare serum levels of soluble forms of interleukin-2 receptor, CD4 and CD8, released by lymphocytes during activation of the immune system, in patients with allergic bronchial asthma, with those in healthy subjects. Significantly higher levels of soluble IL-2R and soluble CD4 were found in patients with asthma compared with the control group. In contrast, lower levels of soluble CD8 values were found in patients with asthma compared to the control group. Significant correlations were found for both sIL-2R and sCD4 and these two molecules, with lung function measured as bronchial responsiveness to inhaled methacholine. These results strengthen previous suggestions that in allergic bronchial asthma, activation of T cells plays a significant role in the disease pathogenesis.**

**Key words:** Allergy, Asthma, sCD4, sCD8, sIL-2R.

### Introduction

It is well known that proliferation of T lymphocytes requires the interaction of a nominal antigen with both accessory cells and T lymphocytes to produce the T cell growth factor interleukin-2 (IL-2). Coincident with T cell activation and production of IL-2, T cells express the IL-2 receptor (IL-2R) which is needed for T-cell proliferation in response to IL-2. Furthermore, activated cells release a truncated form of the α chain of IL-2R (sIL-2R). Various studies have shown a strong association between serum sIL-2R levels and *in vivo* lymphocyte activation, and have indicated that sIL-2R production is directly proportional to cellular IL-2R expression. Because of the small numbers of B cells, NK cells and monocytes expressing cellular IL-2R, it is conceivable that the majority of sIL-2R is elaborated by activated T cells. Hence, serum sIL-2R levels can be used satisfactorily as a marker of T cell activation *in vivo* in patients with malignant, autoimmune and allergic disorders, as well as in subjects affected by systemic infectious diseases or undergoing allograft rejection. Activation of T cells not only leads to the release of sIL-2R, but also causes shedding of other glycoproteins related to T cell surface proteins, including soluble forms of CD4 (sCD4) and CD8 (sCD8). Thus, increased amounts of the soluble forms of these glycoproteins may also be found in the serum in states of increased activation of the immune system.[1–7]

In allergic diseases, the increase of these serum soluble molecules is likely to indicate the immune system activation in response to an allergen challenge. In particular, the levels of serum sCD4 are raised in these diseases where there is a prominent role of helper T (Th) cells.[8–10] In the present paper, we studied the serum levels of sIL-2R, sCD4 and sCD8 as markers of immune activation in asthmatic patients with regard to their clinical status measured as bronchial responsiveness to inhaled methacholine.

### Patients and Methods

*Sample population:* A total of 50 subjects were studied; 25 were patients with bronchial asthma (15 women and ten men, range 19 – 54 years). All patients had *Parietaria* sensitization and a baseline forced expiratory volume in 1 s ($FEV_1$) of at least 80% of predicted value, a provocative concentration of inhaled methacholine causing a 20% fall in $FEV_1$ ($PC_{20}$) $\leqslant$ 4 mg/ml. All patients studied used inhaled bronchodilatators when needed and had not been treated with disodium cromoglycate in the preceding 7 days. None of the patients were using oral or inhaled corticosteroids. Inhaled bronchodilator therapy was withheld for 12 h before the study. The controls consisted of 25 healthy adults (16 women and eleven men, range 20 – 50 years). None of these subjects had a history of prolonged disease and none were ill or taking any drug at the time of the study. The patients and the control group were studied during the *Parietaria* pollen season.

© 1995 Rapid Science Publishers

*Lung function measurements:* $FEV_1$ was measured with a Gould 2400 (Gould, Holland) automated system, taking the highest of three successive measurements, provided the difference between measurements was within 100 ml. A methacholine challenge was performed according to the method of Chai *et al.*[11] Increased concentrations were administered with a Mefar (Markos, Monza, Italy) nebulizer. After baseline measurements of $FEV_1$, subjects inhaled five puffs of saline, since that was considered as the control. Subjects then inhaled increasing concentrations of methacholine, ranging from 0.016 to 1.024 mg/ml. $FEV_1$ was measured 90 s after each concentration step. The provocation was terminated when $FEV_1$ fell by at least 20% from the post-saline value.

*Quantification of soluble molecules:* The sera were stored at −70°C until assay. An enzyme immunoassay test was used to quantify sIL-2R, sCD4 and sCD8. The assays were performed with commercially available kits purchased from Cellfree (T Cell Sciences, Inc., Cambridge, MA, USA). All tests were performed according to the manufacturer's instructions as described previously.[7,12,13] Detection limits in our laboratory for sIL-2R, sCD4 and sCD8 were 50 U/ml; 12 U/ml; and 50 U/ml, respectively.

*Statistical analysis:* All data were expressed as means ± S.D. Correlations were calculated by linear regression. The values for the different groups were compared with Student's *t*-test.

### Results

Patients with allergic asthma had significantly higher levels of sIL-2R and sCD4 than the normal controls (Table 1). As regards soluble forms of CD8, levels were instead significantly decreased in allergic patients compared with the control group (Table 1). According to the simultaneous increase of both sIL-2R and sCD4, a significant correlation was shown between the levels of these two molecules (Table 2).

To assess the role played by activated T lymphocytes in the pathogenesis of asthma, the con-

Table 1. Serum levels (mean ± S.D.) of soluble IL-2R, CD4, and CD8 in 25 normal controls and 25 asthmatic patients

| | Healthy subjects | Patients |
|---|---|---|
| sIL-2R | 306 ± 24[a] | 1007 ± 101[b] |
| sCD4 | 29 ± 3[c] | 76 ± 4[d] |
| sCD8 | 347 ± 20[e] | 178 ± 8[f] |

For a *vs.* b; c *vs.* d; e *vs.* f, $p = 0.0001$

Table 2. Correlation between serum levels of CD4 and sCD8 and IL-2R levels in 25 patients

| | Correlation coefficient | *p* |
|---|---|---|
| sCD4 | +0.613 | 0.0001 |
| sCD8 | −0.307 | N.S. |

centrations of their products in the peripheral blood were correlated with changes in the objective measurements of airway obstruction. Figs. 1 and 2, respectively, demonstrate that bronchial responsiveness (measured as response to inhaled methacholine) correlated with the sIL-2R and sCD4 serum levels.

### Discussion

The sIL-2R, sCD4 and sCD8 glycoproteins are found in relatively large amounts in the circulation and it is possible to obtain normal ranges

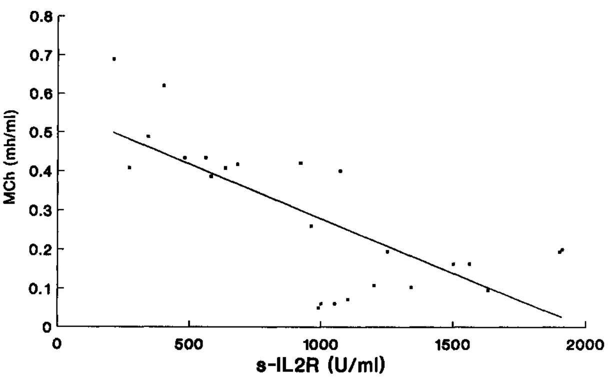

FIG. 1. Correlation between serum sIL-2R values (U/ml) and inhaled concentration of methacholine (MCh) (mg/ml) that determined $FEV_1$ fell by at least 20% from the post-saline value (see Patients and Methods) in 25 asthmatic patients. ($r = -0.751$; $p = 0.00002$).

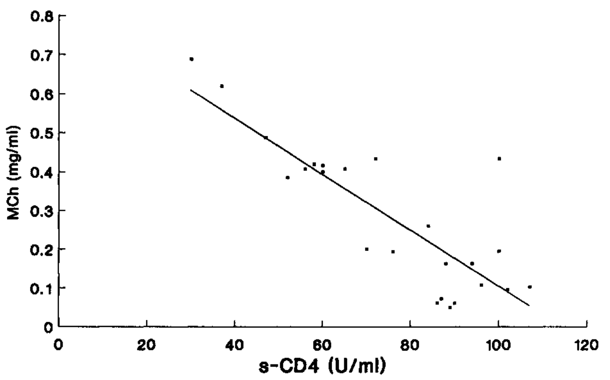

FIG. 2. Correlation between serum sCD4 values (U/ml) and inhaled concentration of methacholine (MCh) (mg/ml) that determined $FEV_1$ fell by at least 20% from the post-saline value (see Patients and Methods) in 25 asthmatic patients. ($r = -0.833$; $p = 0.000001$).

for normal people with upper limits. Their blood levels depend on the number of producing cells and on the number of molecules per cell, and therefore their blood values represent an index of the number and functional state of the producing cells. Thus, their release appears to be a sensitive and quantitative marker of circulating lymphocyte or mononuclear cell activation and may also reflect immune activation in other tissues or fluid compartments.[1–7]

Much attention has been paid recently to the inflammation infiltrate within the airways of patients with asthma. Such inflammation may be the basis of the bronchial hyperreactivity that is the hallmark of this disease. Activated lymphocytes are prominent among the inflammatory cells seen infiltrating the bronchi, and flow cytometric analysis of cells in bronchoalveolar lavage fluid from patients with asthma reveals increased expression of CD25 by CD4 positive lymphocytes. The degree of activation of CD4 positive lymphocytes correlates with the degree of bronchial hyperresponsiveness. In these cells, an activation of the interleukin-3, -4 and -5 and granulocyte-macrophage colony-stimulating factor gene cluster has been observed, a pattern compatible with predominant activation of the Th2 like cell population. Thus, bronchial inflammation in asthma should depend on the activation of Th2 cells that elaborate proinflammatory cytokines involved in the pathogenesis of tissue damage.[8,10,14]

In asthmatic patients, various studies have shown abnormalities of blood immunoregulatory T cells (i.e., the reduction of CD8 cells with an increase of the peripheral CD4/CD8 ratio and the decreased capacity of concanavalin A-stimulated cells to induce suppression *in vitro*). Activated CD4 cells have been observed in the peripheral blood of patients and the number of activated T cells correlates with the airflow obstruction assessed by peak flow measurements. Furthermore, the percentage of activated cells decreases after therapy and clinical improvement. CD8 cells show no evidence of activation. This CD4 cell activation has been shown to be accompanied by significantly elevated serum concentrations of two proteins elaborated by such activated cells, the cytokine interferon-γ and the sIL-2R. A significant relationship has been observed between the sIL-2R level and the degree of airflow obstruction. These observations strongly suggest that CD4 activation is relevant to the pathogenesis of asthma.[8,10,15,16]

Bronchial hyperresponsiveness is a prominent feature in asthma. In our study we have observed a significant inverse relationship between T cell activation and bronchial responsiveness, measured as the responsiveness to inhaled methacholine in our subjects, confirming and extending previous reports.[10,15,16] We have quantified CD4 activation by measuring soluble products released either by blood or by bronchial lymphocytes. This has allowed us to demonstrate a close correlation between the increase of sCD4 and sIL-2R. Our results also demonstrate a significant decrease of sCD8. On the whole, the present findings strengthen the suggestion that in asthma there is an imbalance of the CD4/CD8 ratio and that T helper lymphocytes are the principal determinant for the development of bronchial hyperresponsiveness.[8,14]

### References

1. Caruso C, Candore G, Cigna D, Colucci AT, Modica MA. Biological significance of soluble IL-2 receptor. *Mediators of Inflammation* 1993; **2:** 3–21.
2. Tomkinson BE, Brown MC, Ip SH, Carrabis S, Sullivan JL. Soluble CD8 during T cell activation. *J Immunol* 1989; **142:** 2230–2236.
3. Symons JA, McCulloch JF, Wood NC, Duff GW. Soluble CD4 in patients with rheumatoid arthritis and osteoarthritis. *Clin Immunol Immunopathol* 1991; **60:** 72–82.
4. Symons JA, Wood NC, Di Giovine FS, Duff GW. Soluble CD8 in patients with rheumatic diseases. *Clin Exp Immunol* 1990; **80:** 354–359.
5. Zielinski CC, Pesau B, Muller Ch. Soluble Interleukin-2 receptor and soluble CD8 antigen in active rheumatoid arthritis. *Clin Immunol Immunopathol* 1990; **57:** 74–82.
6. Bresson-Hadni S, Monnot-Jacquard B, Racadot E, Lenys D, Miguet JP, Vuitton DA. Soluble IL-2-receptor and CD8 in the serum and the periparasiatic granuloma of patients with alveolar echinococcosis. *Eur Cytokine Network* 1991; **2:** 339–344.
7. Caruso C, Candore G, Cigna D, *et al.* Serum levels of soluble IL-2R, CD4 and CD8 in chronic active HCV positive hepatitis. *Mediators of Inflammation* 1994; **3:** 185–187.
8. Corrigan CJ, Kay AB. CD4 T-lymphocyte activation in acute severe asthma. *Am Rev Respir Dis* 1990; **141:** 970–977.
9. Walker C, Kagi MK, Ingold P, *et al.* Atopic dermatitis: Correlation of peripheral blood T cell activation, eosinophilia and serum factors with clinical severity. *Clin Exp Allergy* 1993; **23:** 145–153.
10. Virhcow JC, Oehling A, Boer L, *et al.* Pulmonary function, activated T cells, peripheral blood eosinophilia, and serum activity for eosinophil survival *in vitro*: A longitudinal study in bronchial asthma. *J Allergy Clin Immunol* 1994; **94:** 240–249.
11. Chai H, Farr RS, Froehlic LA, *et al.* Standardization of bronchial inhalation challenge procedures. *J Allergy Clin Immunol* 1975; **56:** 323–327.
12. Giordano C, Pantò F, Caruso C, *et al.* Interleukin 2 and soluble interleukin 2 receptor secretion defect *in vitro* in newly diagnosed type I diabetic patients. *Diabetes* 1989; **38:** 310–315.
13. Candore G, Cigna D, Gervasi F, Colucci AT, Modica MA, Caruso C. *In vitro* cytokine production by HLA-B8,DR3 positive subjects. *Autoimmunity* 1994; **18:** 121–132.
14. Robinson DS, Hamid Q, Ying S, *et al.* Predominant TH2-like bronchoalveolar T-lymphocyte population in atopic asthma. *N Engl J Med* 1992; **326:** 298–304.
15. Matsumoto T, Miike T, Yamaguchi K, Murakami M, Kawabe T, Yodoi J. Serum levels of soluble IL-2 receptor, IL-4 and IgE-binding factors in childhood allergic disease. *Clin Exp Immunol* 1991; **85:** 288–292.
16. Lai CKW, Chan CHS, Leung JCK, Lai K. Serum concentration of soluble interleukin 2 receptor in asthma. Correlation with disease activity. *Chest* 1993; **103:** 782–786.

ACKNOWLEDGEMENTS. This work was supported by a grant from the Ministero dell'Università e della Ricerca Scientifica e Tecnologica (60%) to Dr Gabriele Di Lorenzo. The authors wish to thank Dr Antonio T. Colucci for helpful suggestions.

**Received 29 March 1995; accepted 10 April 1995.**

## References

[PDF_00246] Bresson-Hadni S., Monnot-Jacquard B., Racadot E., Lenys D., Miguet J. P., Vuitton D. A. (1991). Soluble IL-2-receptor and CD8 in the serum and the periparasitic granuloma of patients with alveolar echinococcosis.. Eur Cytokine Netw.

[PDF_00267] Candore G., Cigna D., Gervasi F., Colucci A. T., Modica M. A., Caruso C. (1994). In vitro cytokine production by HLA-B8,DR3 positive subjects.. Autoimmunity.

[PDF_00233] Caruso C., Candore G., Cigna D., Colucci A. T., Modica M. A. (1993). Biological significance of soluble IL-2 receptor.. Mediators Inflamm.

[PDF_00250] Caruso C., Candore G., Cigna D., Tripi S., Di Gaetano G., Migneco G., Montalto G., Ruggieri I., Notarbartolo A. (1994). Serum Levels of Soluble IL-2R, CD4 and CD8 in Chronic Active HCV Positive Hepatitis.. Mediators Inflamm.

[PDF_00253] Corrigan C. J., Kay A. B. (1990). CD4 T-lymphocyte activation in acute severe asthma. Relationship to disease severity and atopic status.. Am Rev Respir Dis.

[PDF_00262] Giordano C., Pantò F., Caruso C., Modica M. A., Zambito A. M., Sapienza N., Amato M. P., Galluzzo A. (1989). Interleukin 2 and soluble interleukin 2-receptor secretion defect in vitro in newly diagnosed type I diabetic patients.. Diabetes.

[PDF_00273] Lai C. K., Chan C. H., Leung J. C., Lai K. N. (1993). Serum concentration of soluble interleukin 2 receptors in asthma. Correlation with disease activity.. Chest.

[PDF_00270] Robinson D. S., Hamid Q., Ying S., Tsicopoulos A., Barkans J., Bentley A. M., Corrigan C., Durham S. R., Kay A. B. (1992). Predominant TH2-like bronchoalveolar T-lymphocyte population in atopic asthma.. N Engl J Med.

[PDF_00236] Symons J. A., McCulloch J. F., Wood N. C., Duff G. W. (1991). Soluble CD4 in patients with rheumatoid arthritis and osteoarthritis.. Clin Immunol Immunopathol.

[PDF_00255] Walker C., Kägi M. K., Ingold P., Braun P., Blaser K., Bruijnzeel-Koomen C. A., Wüthrich B. (1993). Atopic dermatitis: correlation of peripheral blood T cell activation, eosinophilia and serum factors with clinical severity.. Clin Exp Allergy.

